# Quality of life of mothers of children with Down syndrome: a study comparing those with and without hearing loss

**DOI:** 10.3389/fpsyt.2025.1611074

**Published:** 2025-08-28

**Authors:** Rania Alkahtani, Huny Bakry, Lama M. Al Hawi, Jana D. Alshehri, Reem Elbeltagy

**Affiliations:** ^1^ Department of Health Communication Sciences, College of Health and Rehabilitation Sciences, Princess Nourah bint Abdulrahman University, Riyadh, Saudi Arabia; ^2^ Department of Health Sciences, College of Health and Rehabilitation Sciences, Princess Nourah bint Abdulrahman University, Riyadh, Saudi Arabia

**Keywords:** Down syndrome, hearing loss, quality of life, mothers, children

## Abstract

**Background:**

Down syndrome is a genetic disorder present from birth, leading to various physical and cognitive challenges. Some children with Down syndrome also experience hearing loss. The combined impact of raising a child with Down syndrome and hearing loss can affect the quality of life (QoL) of mothers. The aim of this study was to examine the differences in QoL between mothers of children with Down syndrome who have hearing loss and those without hearing loss.

**Methods:**

A cross-sectional study was conducted with 103 mothers of children with Down syndrome. Data were collected using the Arabic version of the WHOQOL-BREF, which assesses the QoL across four domains, including physical health, psychological health, social relationships, and environment, with scores ranging from 0-100.

**Results:**

In the studied sample, 16.5% of mothers reported that their child had hearing loss. The mean QoL scores for the total sample were 65.8 ± 18.6 in physical health, 72.6 ± 17.5 in psychological health, 65.6 ± 13.7 in social relationships, and 68.7 ± 16.8 in environment. There were no significant differences in QoL scores across any domain between mothers of children with and without hearing loss (p > 0.05). Mothers’ perceptions of their overall QoL and health were high and similar between both groups.

**Conclusion:**

Most mothers in this study reported satisfactory QoL. While HL does not seem to drastically affect overall QoL across various domains, it is evident that social challenges persist.

## 1 Introduction

Down syndrome (DS) is the most prevalent chromosomal disorder. Children diagnosed with DS typically exhibit slower developmental rates and a range of other health problems, including congenital heart disease, endocrine disorders, eye disorders, and obstructive sleep apnea (1). These conditions necessitate heightened parental dedication, impacting the entire family dynamics (2).

Children with DS commonly experience recurrent ear infections and hearing loss (HL), with a reported prevalence of 47% (3). HL in children with DS may have various etiologies. Conductive hearing loss (CHL), the most common type of HL in these children, is often caused by factors such as recurrent wax impaction due to stenosis in the external auditory canal, recurrent otitis media from Eustachian tube dysfunction, and ossicular anomalies in the middle ear (4). Sensorineural hearing loss (SNHL), which is less common in children with DS, can result from abnormalities in the inner ear, such as inner ear hypoplasia, cochlear dysplasia, or cochlear nerve canal anomalies (4). Additionally, SNHL may be influenced by perinatal risk factors or remain idiopathic (3).

Mothers of newly diagnosed children with HL often grapple with persistent feelings of being overwhelmed and inadequately equipped to manage their child’s HL effectively, which may manifest as anger (5). Existing literature has shown that mothers of children with DS alone often experience reduced QoL due to increased caregiving demands and concerns about long-term developmental outcomes (6). Similarly, mothers of children with HL alone report emotional stress, social isolation, and challenges accessing early intervention services (7) However, limited research has explored how the co-occurrence of DS and HL may uniquely impact maternal QoL, despite evidence that both conditions independently impose significant caregiving burdens.

When DS is coupled with HL, the challenges can be further compounded. Communication difficulties may be more pronounced, and accessing appropriate interventions, addressing increased social isolation, and ensuring adequate educational advocacy and support services may require additional effort. Mothers may experience heightened levels of stress and anxiety related to societal stigma and concerns about their child’s ability to communicate effectively and participate fully in social and educational settings (8, 9).

Given the specialized attention required by children with DS, mothers often find themselves involved in every aspect of their child’s activities, which might affect their quality of life (QoL). The World Health Organization (WHO) defined the QoL as the individuals’ perceptions of their position in life within the context of their cultures, values, and goals. It is a subjective, multidimensional construct that encompasses both positive and negative elements of evaluation (10).

The present study aimed to assess the QoL among mothers of children with DS, whether their children experience HL or not, to explore if HL adds more burden to their QoL. By examining whether HL further exacerbates maternal burden, the current study aims to fill a critical gap in understanding how dual diagnoses affect maternal well-being. The study distinguishes itself from existing literature through the following. First, while extensive literature exists on the impact of either DS or HL on family well-being, the specific challenges and their combined effect on maternal QoL when these conditions co-occur remain underexplored except by Hussin et al. (11). Second, it specifically examined mothers in Saudi Arabia. This provided an underrepresented perspective on how specific Saudi cultural factors (e.g., family structures, local resources) uniquely influence maternal QoL. Third, this study explored additional variables such as the relationship between QoL and maternal/child age, offering a broader view of influencing factors than prior research. Previous studies have suggested that a caregiver’s age may influence coping strategies, stress tolerance, and access to social support, all of which can impact QoL (12). Similarly, the age of the child may reflect different caregiving demands such as early intervention needs in younger children versus long-term planning stress in older children. Including these variables provides a broader understanding of potential factors influencing maternal QoL in the context of raising a child with DS.

By addressing these specific gaps, this study enhanced the understanding of complex caregiving demands and provided insights for targeted support interventions for mothers of children with DS and co-occurring HL in this particular cultural setting.

## 2 Materials and methods

A cross-sectional study was conducted, including 103 mothers of children with DS. Given the inherent rarity of DS (1.8:1000 in Saudi Arabia and 1:700–1000 globally), achieving a sample size of 103 represents a substantial cohort for this population (13). Participants were recruited through the Down Syndrome Charity in Saudi Arabia. Additionally, recruitment was conducted via social media platforms such as X, WhatsApp, and Telegram to broaden the search area for participants.

Data were collected using the Arabic version of the WHOQOL-BREF (10). The questionnaire comprises 26 questions, including two questions about overall QoL and general health, and 24 questions representing specific facets from the original WHOQOL-100 tool (14). The four domains assessed are physical health, psychological health, social relationships, and environment with scores ranging from 0-100; higher scores indicate better QoL. Categorization of QoL was based on a 60% cutoff, where scores ≥ 60% were considered “good”, and scores < 60% were considered “poor” (15).

The WHOQOL-BREF was chosen to assess maternal QoL due to its comprehensive and holistic approach, covering physical, psychological, social, and environmental health, which is crucial for understanding the multifaceted impact of caregiving. Its proven cross-cultural validity, developed through international collaboration and specifically validated in Arabic-speaking populations (16), makes it highly suitable for the study’s population. The instrument’s brevity (26 items) minimizes respondent burden for busy caregivers, improving data quality and feasibility. Furthermore, the WHOQOL-BREF possesses established psychometric properties and has been widely validated and utilized in various caregiver populations, including parents of children with chronic conditions and disabilities. This allows for meaningful comparisons not only between the study groups (mothers of children with DS with vs. without HL) but also with broader population norms. While specialized caregiver tools exist, the WHOQOL-BREF’s generic nature provides a robust and broadly comparable measure of overall QoL. It was selected over caregiver-specific HRQOL tools because of its strong psychometric foundation, extensive cross-population validation, and its ability to facilitate comparison with both clinical and general population norms, features particularly important for the aims of this study.

### 2.1 Participants inclusion and exclusion criteria

**Inclusion criteria:** Mothers of children with DS who reside in Saudi Arabia and are willing to participate in the study by providing informed consent.

**Exclusion Criteria:**

- Mothers with a diagnosed mental health condition (e.g., severe depression, anxiety disorder, or psychosis) that could significantly impact their self-reported QoL, or other chronic illnesses that might heavily influence their QoL independent of their child’s condition. This helps ensure that the measured QoL is primarily related to the caregiving experience for a child with DS.
- Children with DS who have additional significant co-morbidities beyond DS and HL (e.g., severe congenital heart defects requiring ongoing intensive care, cerebral palsy, or autism spectrum disorder). This helps to isolate the impact of DS and HL.
- If the child with DS is not currently living at home with the mother (e.g., institutionalized, living independently), this would significantly alter the daily caregiving experience.
- Inability or unwillingness to provide informed consent.
- Inability to communicate effectively in the study language.

The final sample included 103 mothers of children with DS who were residents in Saudi Arabia and provided informed consent. Among them, 17 mothers reported that their child had co-occurring HL (DS+HL), while 86 mothers reported no known HL (DS-only). Mothers were selected as participants due to their primary caregiving role in the Saudi cultural context, which typically involves direct responsibility for managing the child’s medical, developmental, and emotional needs.

HL status was identified based on maternal report, wherein participants were asked whether a healthcare professional had diagnosed their child with HL. No audiological testing was conducted as part of the study. Some demographic variables, such as maternal education level, employment status, and household income were not collected in this study, which limits the ability to explore how these factors may influence QoL outcomes.

### 2.2 Data analysis

Statistical analysis was performed using IBM SPSS version 29. Descriptive statistics were presented as frequencies, percentages, means, and standard deviations (SD). Differences between means were evaluated using the Welch’s t-test, which is appropriate when unequal variances and unequal group sizes are present. This test was selected to account for the imbalance in sample size between the two groups (DS-only vs. DS+HL). Pearson correlation was utilized to determine relationships between quantitative variables. A p-value of less than 0.05 was considered statistically significant.

### 2.3 Ethical consideration

Ethical approval was obtained from the Institutional Review Board (ID 22-0937). Participants consents were obtained electronically.

## 3 Results

The demographic characteristics of the studied sample including the age of the mother, the age of the child and the gender of the child are detailed in **Table 1**. Among the total children, 16.5% (n=17) had HL and 22.3% (n=23) had recurrent ear infection as reported by their mothers. Of those with HL, 11.8% had SNHL, 23.5% had CHL and 64.7% were unsure of the type of HL their child experienced.

**Table 1:** Demographic characteristics of the studied sample (n= 103).

Demographic characteristics	No.	%
Mother’s age (years)		
18-25	8	7.8
26-35	10	9.7
36-45	38	36.9
46-55	34	33
≥56	13	12.6
Child’s gender		
Male	56	54.5
Female	47	45.6
Child’s age (years)		
Less than 1	8	7.8
01-May	27	26.2
06-Oct	22	21.4
Nov-15	25	24.3
≥16	21	20.4
Total	103	100

The mean QoL scores among the studied sample in the physical, psychological, social and environmental domains were 65.8 ± 18.6, 72.6 ± 17.5, 65.6 ± 13.7 and 68.7 ± 16.8 respectively (**Table 2**). Most participants reported good QoL in the physical, psychological, and environmental domains regardless of their child’s hearing status, with ≤30% reporting poor QoL in these domains (**Table 3**). However, slightly over half of the mothers of children with both DS and HL reported poor QoL scores in the social domain (53%) compared to 29.1% of the mothers of children with DS but without HL (**Table 3**).

**Table 2:** Means and SDs of the scores of the QoL domains among the total studied sample (n= 103).

QoL domain	Mean ± SD
Physical health	65.8 ± 18.6
Psychological health	72.6 ± 17.5
Social relationships	65.6 ± 13.7
Environment	68.7 ± 16.8
Total score	68.2 ± 16.6

**Table 3:** Mothers’ perception of their QoL of each domain (n= 103).

Mothers’ perception	Mothers of children with DS who experience HL No (%)	Mothers of children with DS who do not experience HL No (%)
Physical health		
Good	12 (70.5)	62 (72)
Poor	5 (29.5)	24 (28)
Psychological health		
Good	15 (88.2)	62 (72)
Poor	2 (11.8)	24 (28)
Social relationships		
Good	8 (47)	61 (70.9)
Poor	9 (53)	25 (29.1)
Environment		
Good	12 (70.5)	60 (69.8)
Poor	5 (29.5)	26 (30.2)

To determine if HL poses more challenge to the QoL of mother of children with DS, the differences in mean scores across different QoL domains between mothers of children with HL and those without HL were assessed using the Welch’s t-test. The results revealed no significant differences in any of the domains (**Table 4**).

**Table 4:** Means and SDs of the scores of the QoL domains among mothers of children of both groups (with and without HL) (n= 103).

QoL domain	Mothers of children with DS who experience HL (Mean ± SD)	Mothers of children with DS who do not experience HL (Mean ± SD)	P value*
Physical health domain	66.7 ± 17.4	65.6 ± 18.9	0.8
Psychological health domain	76.7 ± 15.6	71.8 ± 17.8	0.3
Social relationships domain	59.8 ± 15.6	66.7 ± 13.1	0.06
Environment domain	67.7 ± 13.7	68.9 ± 17.4	0.8
Total	67.7 ± 15.5	68.2 ± 16.8	0.8

*Welch’s t-test.

The majority of mothers reported a good perception of their overall QoL (82.4% for others of children with HL and 88.4% for mothers of children with normal hearing). Similarly, most mothers reported a good perception of their health status (76.5% for mothers of children with HL and 86.1% for mothers of children with normal hearing). The association between mothers’ perceptions of their QoL and health status and whether they had a child with HL or not was examined using the Welch’s t-test, which also revealed no significant association (**Table 5**). Additionally, the correlation between the different QoL domains and both the mother’s age and the child’s age were investigated using the Pearson correlation test, which indicated no significant correlations (**Table 6**).

**Table 5:** Perception of the mothers regarding their overall QoL and health status (n= 103).

Mothers’ perception	Mothers of children with DS who experience HL (Mean ± SD)	Mothers of children with DS who do not experience HL (Mean ± SD)	P value*
Mother’s Perception regarding their overall QOL	4.3 ± 0.8	4.5 ± 0.7	0.4
Mother’s Perception regarding their overall health status	4.4 ± 0.9	4.2 ± 0.8	0.4

*Welch’s t-test.

**Table 6:** Correlation between QoL domains of mothers of children with DS who experience HL and both the age of the mother and the age of the child.

QoL domain	Mother’s age		Child’s age	
	r*	P value	r*	P value
Mother’s Perception regarding their overall QOL	-0.09	0.7	0.1	0.6
Mother’s Perception regarding their overall health status	-0.3	0.3	-0.02	0.9
Physical health domain	-0.1	0.6	-0.05	0.8
Psychological health domain	-0.2	0.4	0.06	0.8
Social relationships domain	-0.3	0.3	-0.3	0.2
Environment domain	0.04	0.9	0.2	0.4

*Pearson correlation test.

## 4 Discussion

Mothers play a crucial role in the lives of their children, and this is especially true for children with disabilities, such as DS. Studies have shown that intellectual disabilities and other disorders lead to significant socio-occupational dysfunction and impaired QoL for caregivers (17). Children with DS may also experience HL (3), adding an additional burden to their mothers, who may face unique communication challenges and require additional support services to ensure their child’s well-being (8, 9). Caregivers often report physical difficulties, such as insufficient sleep, lack of exercise, irregular and inadequate meals, and neglecting their own medical needs. These challenges can result in clinical depression, social isolation, high stress, and low QoL (18). Therefore, it is important to assess the QoL of mothers of children with disabilities to provide them with better support and interventions, if needed.

The findings of the current study indicate that the majority of mothers reported satisfactory QoL, aligning with results from other studies (11, 19, 20) and even surpassing some (6, 21). However, the social domain had the lowest score, particularly for mothers of children with HL. This may be due to social isolation stemming from feelings of difference from other mothers and limited social networks due to communication barriers, which can lead to loneliness (8). Additionally, isolation could be exacerbated by limited support networks or societal misunderstandings about their children’s conditions (22).

The present study found the psychological domain to have the highest QoL scores, while the social domain was the lowest, a pattern that contrasts with findings from Hussin et al. (11), where the social domain scored better. This notable discrepancy may be explained by several contextual and methodological differences. While previous studies have shown similar findings (19), others reported higher social domain scores (11, 23, 24), attributing this to strong parent-child relationships and familial bonds developed through shared experiences. The differing QoL domain scores between the current study and Hussin et al. (11) can be significantly attributed to cultural and contextual influences. The availability and nature of formal and informal support systems (healthcare, social programs) in each respective context profoundly influence how caregivers experience and perceive their psychological well-being and social connections.

Additionally, the current study did not assess marital status, education level, and income, which presents a limitation when comparing our findings to Hussin et al. (11). Socioeconomic status and education are known to influence access to resources, coping mechanisms, and social networks, thereby impacting perceived QoL. Similarly, marital status and the availability of family support significantly affect psychological well-being (by reducing isolation and stress) and social domain scores. These unmeasured differences in participant demographics and family structures likely contributed to the observed variations in QoL domain scores between the two studies.

The results suggest that HL in children with DS may not directly impact the overall QoL of their mothers across various domains. This aligns with Hussin et al. (11), who found no significant difference in QoL between mothers of children with DS and HL and those with DS alone, with 60% of mothers reporting satisfaction. This could be due to the typically mild to moderate nature of HL in DS cases and the ability of some children with mild HL to compensate (24).

Although no significant statistical difference was found between mothers of children with HL and those without in the social domain, the difference is nonetheless noteworthy. More than half of the mothers of children with HL (53%) reported poor scores in this domain compared to 29% of mothers of children without HL. This nearly twofold disparity, while not statistically significant, suggests that the co-occurrence of DS and HL may uniquely and substantially burden a mother’s social life.

This burden may stem from multiple interrelated factors. Compounded communication challenges can increase time and resource demands on the mother, while limited social opportunities for the child may further restrict the mother’s own social engagement. In addition, mothers of children with both conditions may face greater social isolation due to a perceived lack of understanding from single-diagnosis support networks and the presence of societal stigma.

These challenges may be further intensified by the cumulative stress of managing multiple diagnoses, navigating early intervention services, advocating for educational placements, and coordinating ongoing therapy and communication support. Emotional strain, financial pressures, and social stigma could all contribute to the reduced QoL (25) reported by these mothers. Taken together, these findings underscore the importance of the social domain as a critical area for future targeted interventions and research, even if broader QoL differences were not statistically significant.

Contrary to previous studies suggesting that behavioral problems in children with DS increase with age, which could decrease caregivers’ QoL (26, 27), the current study found no significant correlation between the child’s age and different QoL domains. However, the result is consistent with other studies such as Vadakedom et al. (6). The correlation between mothers’ age and QoL is also debated in the literature, with some studies showing negative correlations (20), others showing positive correlations (27), and some showing no correlation (19). Our study supports those reporting no correlation (19), suggesting that mothers of all ages adapt well to their children’s conditions, particularly since children with DS often exhibit fewer behavioral problems compared to those with other disabilities (28).

The study has some limitations. The cross-sectional design restricts causal inferences, and the reliance on maternal reports rather than objective audiological assessments could affect the accuracy of the reported prevalence of HL. This approach may result in misclassification or underreporting of HL, especially in cases where hearing issues have not been formally diagnosed. The absence of audiological confirmation also limits our ability to assess the severity and type of HL, which may have differential impacts on maternal QoL. Additionally, potential confounding factors such as the severity of the child’s HL, maternal support systems, and socioeconomic status were not explored. Furthermore, the notable imbalance in sample sizes between the DS-only and DS+HL groups may have limited the statistical power to detect significant differences between groups. While the use of Welch’s t-test helped to partially account for this issue, the small number of participants in the DS+HL group (n=17) remains a limitation that may affect the robustness and generalizability of between-group comparisons. Future studies with larger and matched samples are recommended to validate these findings.

## 5 Conclusions and recommendations

Most mothers in this study reported satisfactory QoL. Notably, the social domain had the lowest scores, particularly for mothers of children with HL. This suggests that social isolation and communication barriers may significantly impact QoL. While HL does not seem to drastically affect overall QoL across various domains, it is evident that social challenges persist.

The study found no significant correlations between the child’s or mother’s age and QoL, indicating that age might not be a critical factor in determining QoL in this context. This suggests that other variables may be more influential in shaping QoL for mothers of children with DS.

To improve the accuracy of findings, longitudinal studies are needed to explore how HL and DS interact over time and how QoL evolves as children with DS grow older. Such research could offer valuable insights into the long-term effects of HL and other factors on mothers’ QoL. Further investigation into the role of maternal support systems and societal understanding is crucial. Understanding these factors could inform the development of targeted support mechanisms to alleviate social isolation and stigma. Importantly, there is a pressing need for structured interventions specifically addressing the social QoL of mothers caring for children with both DS and HL. These may include accessible community-based programs, social engagement initiatives, and tailored peer support networks that can help mitigate isolation and emotional burden.

A focused analysis of the social domain of QoL is warranted. Research should examine specific aspects of social isolation and communication barriers faced by mothers of children with HL. Developing interventions to enhance social integration and support networks could improve QoL for these mothers. Prioritizing this subgroup in intervention design is essential, as they may face compounded challenges due to dual diagnoses. Additionally, future studies should consider socioeconomic status and other potential confounders to provide a comprehensive understanding of the factors influencing QoL. This broader perspective could help tailor support strategies more effectively.

## Data Availability

The original contributions presented in the study are included in the article/supplementary material. Further inquiries can be directed to the corresponding author.

## References

[1] Centers of Disease Control and Prevention. Data Statistics on Down Syndrome (2023). Available online at: https://www.cdc.gov/birth-defects/living-with-down-syndrome/?CDC_AAref_Val=https://www.cdc.gov/ncbddd/birthdefects/downsyndrome/data.html (Accessed August 14, 2024).

[2] Bourke J Ricciardo B Bebbington A Aiberti K Jacoby P Dyke P. Physical and mental health in mothers of children with Down syndrome. J Pediatr. (2008) 153:320–6. doi: 10.1016/j.jpeds.2008.02.047, PMID: 18534233 PMC2586647

[3] De Schrijver L Topsakal V Wojciechowski M Van de Heyning P Boudewyns A. Prevalence and etiology of sensorineural hearing loss in children with down syndrome: A cross-sectional study. Int J Pediatr Otorhinolaryngol. (2019) 116:168–72. doi: 10.1016/j.ijporl.2018.10.048, PMID: 30554691

[4] Shott SR. Down syndrome: common otolaryngologic manifestations. Am J Med Genet C Semin Med Genet. (2006) 142C:131–40. doi: 10.1002/ajmg.c.30095, PMID: 16838306

[5] Kurtzer-White E Luterman D. Families and children with hearing loss: Grief and coping. Ment Retard Dev Disabil Res Rev. (2003) 9:232–5. doi: 10.1002/mrdd.10085, PMID: 14648815

[6] Vadakedom SS Antony JM Padma BK Mammen DS Thankappan BP. Quality of life of mothers of children with Down syndrome. J Evol Med dent Sci. (2017) 6:2939–43. doi: 10.14260/Jemds/2017/633

[7] Pelarak F Radmehr M Sanjari H Abdalvand A Forouzesh F. The children with hearing disabilities: Mothers’ general health status and children’s quality of life. JundishapurJChronicDisCare. (2021) 10:e115472. doi: 10.5812/jjcdc.115472

[8] Gunjawate DR Ravi R Driscoll C. Stress among parents of children with hearing loss and how they deal with it: A systematic review. Int Arch Otorhinolaryngol. (2023) 27:166–77. doi: 10.1055/s-0042-1743273, PMID: 36714900 PMC9879637

[9] Watanabe M Kibe C Sugawara M Miyake H. Courtesy stigma of parents of children with Down syndrome: Adaptation process and transcendent stage. J Genet Couns. (2022) 31(3):746–57. doi: 10.1002/jgc4.1541, PMID: 34951509 PMC9415099

[10] The WHOQOL GROUP. Development of the World Health Organization WHOQOL-BREF quality of life assessment. The WHOQOL Group. Psychol Med. (1998) 28:551–8. doi: 10.1017/s0033291798006667, PMID: 9626712

[11] Hussin N Ismail A Ismail J Abdullah A. The quality of life of mothers of down syndrome children with and without hearing impairment in Universiti Kebangsaan Malaysia Medical Center. Indian J Otol. (2021) 27:189–92. doi: 10.4103/indianjotol.INDIANJOTOL_70_20

[12] Alrayes N Issa NM Alghubayshi OY Al-Amaa JY Alsabban AH Al Shaer DS. Quality of life in children with Down syndrome and its association with parent and child demographic characteristics: Parent-reported measures. Mol Genet Genomic Med. (2024) 12:e2337. doi: 10.1002/mgg3.2337, PMID: 38093585 PMC10767681

[13] Parveen S Ahmad A Reshi AA. Empowering lives: navigating the landscape of down syndrome support in Saudi Arabia. JDR. (2024) 3. doi: 10.57197/JDR-2024-0044

[14] The WHOQOL GROUP. The World Health Organization Quality of Life assessment (WHOQOL): position paper from the World Health Organization. Soc Sci Med. (1995) 41:1403–9. doi: 10.1016/0277-9536(95)00112-k, PMID: 8560308

[15] Silva PA Soares SM Santos JF Silva LB. Cut-off point for WHOQOL-bref as a measure of quality of life of older adults. Rev saude publica. (2014) 48:390–7. doi: 10.1590/s0034-8910.2014048004912, PMID: 25119934 PMC4203073

[16] Dalky HF Meininger JC Al-Ali NM. The reliability and validity of the arabic world health organization quality of life-BREF instrument among family caregivers of relatives with psychiatric illnesses in Jordan. J Nurs Res. (2017) 25:224–30. doi: 10.1097/JNR.0000000000000146, PMID: 28481818

[17] Malhotra S Khan W Bhatia MS. Quality of life of parents having children with developmental disabilities. Delhi Psychiatry J. (2012) 15:171–6.

[18] Dumas EE Wolf LC Fisman SN Culligan A. Parenting stress, child behavior problems, and dysphoria in parents of children with autism, Down syndrome, behavior disorders, and normal development. Exceptionality J Spec Educ. (1991) 2:97–110. doi: 10.1080/09362839109524770

[19] AlAhmari FS Alageel AF Aldosari MA Bagha MY. The quality of life of parents of children with Down syndrome in a tertiary care hospital: a qualitative research study at Saudi Arabia. Ann Intern Med. (2022) 81:104428. doi: 10.1016/j.amsu.2022.104428, PMID: 36147136 PMC9486666

[20] Geok CK Abdullah KL Kee LH. Quality of life among Malaysian mothers with a child with Down syndrome. Int J Nurs Pract. (2013) 19:381–9. doi: 10.1111/ijn.2013.19.issue-4, PMID: 23915407

[21] Senses Dinc G Cop E Tos T Sari E Senel S. Mothers of 0–3-year-old children with Down syndrome: Effects on quality of life. Pediatr Int. (2019) 61:865–71. doi: 10.1111/ped.13936, PMID: 31267616

[22] Wright-Sexton LA Compretta CE Blackshear C Henderson CM. Isolation in parents and providers of children with chronic critical illness. Pediatr Crit Care Med. (2020) 21:e530–7. doi: 10.1097/PCC.0000000000002344, PMID: 32195899

[23] Dabrowska AQA Pisula E. Parenting stress and coping styles in mothers and fathers of pre-school children with autism and Down syndrome. J Intellect. (2010) 54:266–80. doi: 10.1111/j.1365-2788.2010.01258.x, PMID: 20146741

[24] Moore DR Zobay OF Erguson MA. Minimal and mild hearing loss in children: Association with auditory perception, cognition, and communication problems. Ear Hear. (2020) 41:720–32. doi: 10.1097/AUD.0000000000000802, PMID: 31633598 PMC7160041

[25] Fucà E Costanzo F Ursumando L Vicari S. Parenting stress in mothers of children and adolescents with down syndrome. J Clin Med. (2022) 11:1188. doi: 10.3390/jcm11051188, PMID: 35268278 PMC8911183

[26] Norizan A Shamsuddin K. Predictors of parenting stress among Malaysian mothers of children with Down syndrome. J Intellect. (2010) 54:992–1003. doi: 10.1111/j.1365-2788.2010.01324.x, PMID: 20868445

[27] Corrice AM Glidden LM. The Down syndrome advantage: fact or fiction? A.J.I.D.D. (2009) 114:254–68. doi: 10.1352/1944-7558-114.4.254-268, PMID: 19642708

[28] Eisenhower AS Baker BL Blacher J. Preschool children with intellectual disability: syndrome specificity, behavior problems, and maternal well-being. J Intellect. (2005) 49:657–71. doi: 10.1111/j.1365-2788.2005.00699.x, PMID: 16108983 PMC3072759

